# Thromboelastometry in Neonates with Respiratory Distress Syndrome: A Pilot Study

**DOI:** 10.3390/diagnostics11111995

**Published:** 2021-10-27

**Authors:** Georgios N. Katsaras, Rozeta Sokou, Andreas G. Tsantes, Aikaterini Konstantinidi, Dimitra Gialamprinou, Daniele Piovani, Stefanos Bonovas, Anastasios G. Kriebardis, Georgios Mitsiakos, Styliani Kokoris, Argirios E. Tsantes

**Affiliations:** 1 Neonatal Intensive Care Unit, Nikaia General Hospital “Aghios Panteleimon”, Nikaia, 18454 Piraeus, Greece; gkatsaras84@gmail.com (G.N.K.); sokourozeta@yahoo.gr (R.S.); kmaronia@gmail.com (A.K.); 2 Second Department of Neonatology and Neonatal Intensive Care Unit, Papageorgiou General Hospital, School of Medicine, Aristotle University of Thessaloniki, Nea Efkarpia, 56403 Thessaloniki, Greece; gialamprinou@gmail.com (D.G.); mitsiakos@auth.gr (G.M.); 3 Laboratory of Haematology and Blood Bank Unit, Attikon University Hospital, School of Medicine, National and Kapodistrian University of Athens, Haidari, 12462 Athens, Greece; andreas.tsantes@yahoo.com (A.G.T.); stelkok19@gmail.com (S.K.); 4 Department of Biomedical Sciences, Humanitas University, Via Rita Levi Montalcini 4, Pieve Emanuele, 20090 Milan, Italy; dpiovani@hotmail.com (D.P.); sbonovas@gmail.com (S.B.); 5 IRCCS Humanitas Research Hospital, Via Manzoni 56, Rozzano, 20089 Milan, Italy; 6 Laboratory of Reliability and Quality Control in Laboratory Hematology, Department of Biomedical Science, School of Health and Caring Science, University of West Attica, Egaleo, 12243 Athens, Greece; akrieb@uniwa.gr

**Keywords:** respiratory distress syndrome, infant, newborn, thromboelastometry

## Abstract

Background: Although respiratory distress syndrome (RDS) constitutes a postnatal risk factor for bleeding and thromboembolic events in neonates, few studies have addressed this issue. We aimed to evaluate the hemostatic profile of neonates with RDS using rotational thromboelastometry (ROTEM). Methods: An observational study was conducted from November 2018 to November 2020 in the NICU of General Hospital of Nikaia “Aghios Panteleimon”. Preterm and term neonates with RDS hospitalized in the NICU were included and EXTEM (tissue factor-triggered extrinsic pathway), INTEM (ellagic acid activated intrinsic pathway), and FIBTEM (with platelet inhibitor cytochalasin D) assays were performed at the onset of the disease. Results: A hypocoagulable profile was noted in neonates with RDS compared to controls, expressed as significant prolongation of EXTEM CT (clotting time) and CFT (clot formation time), lower EXTEM A10 (amplitude at 10 min), MCF (maximum clot firmness), and LI60 (lysis index). Furthermore, prolongation of INTEM CFT and FIBTEM CT, and decreased INTEM and FIBTEM A10 and MCF were found in neonates with RDS. Multivariable logistic regression analysis showed that RDS is an independent factor for the recorded alterations in ROTEM variables. Conclusions: RDS is associated with a hypocoagulable profile and greater hyperfibrinolytic potential compared to healthy neonates.

## 1. Introduction

In 1959, Avery and Mead discovered that the pathophysiology of hyaline membrane disease (HMD) involved pulmonary surfactant (PS) deficiency, and consequently, the disease name was changed to respiratory distress syndrome (RDS) [1]. In recent years, some authors have been referring to this syndrome as surfactant deficiency disorder (SDD) [2]. RDS is negatively correlated with gestational age (GA), with the risk for developing RDS increasing as GA decreases [3]. While in preterm neonates RDS develops because of reduced surfactant production, in full-term neonates it develops secondarily by surfactant consumption, due to either a maladaptation to the extra-uterine life, persistent pulmonary hypertension (PPHN), or external adverse events like meconium aspiration [4].

In RDS, inflammatory mediators accumulate in the alveolar space, neutrophils penetrate it, and “glass” membranes are created in the alveolar space from the deposition of fibrin, the final derivative of coagulation [5]. Apart from the activation of the inflammation cascade, RDS is a possible trigger of a coagulative state [6,7]. Even though RDS has been found to activate intravascular coagulation and cause abnormalities in fibrinolysis, few studies have addressed this issue [8,9,10,11]. In microcirculation and alveolar spaces of preterm neonates with severe RDS, fibrin deposits have been found [12]. Further, the elevated thrombin/ antithrombin III (TAT) complexes and reduced antithrombin III (AT) activity seen in neonates with RDS have been correlated with the severity of the disease [7]. On the contrary, a more recent study showed that AT was not affected by RDS [13]. This may be due to surfactant therapy, since its initiation in the 1980s, decreasing the incidence and severity of the disease and thus altering its pathway. Currently, the “ground glass” appearance with air bronchogram and hypo-aeration are rarely seen [14,15,16]. Despite the change of the disease in the past decades, RDS remains worth exploring as a postnatal risk factor for bleeding and thromboembolic events [17,18,19].

Rotational thromboelastometry (ROTEM) is a viscoelastic test that examines the dynamics of blood coagulation, clotting time, clot stabilization, and clot lysis [20]. Limited studies have used ROTEM to evaluate intraoperative hemostasis and estimate the coagulation profile of critically ill neonates, but no studies have been conducted in neonates with RDS [21,22,23,24,25,26]. Taking this into consideration, we aimed to delineate the hemostatic profile of neonates with RDS using ROTEM.

## 2. Materials and Methods

### 2.1. Study Design

We conducted an observational study at the Neonatal Intensive Care Unit (NICU) of Nikaia General Hospital “Aghios Panteleimon”, Piraeus, Greece, over a 2-year period (from November 2018 to November 2020). The protocol of this single-center study was approved by the Institutional Review Board of Nikaia General Hospital (28/11/2018, 24/27), and informed consent was obtained from participants prior to recruitment.

### 2.2. Participants

All term and preterm hospitalized neonates who developed RDS were recruited. For the definition of RDS, the Vermont Oxford definition for RDS in preterm neonates was used: (a) arterial oxygen tension (PaO2) < 50 mm Hg and central cyanosis in room air, (b) requirement for supplemental oxygen to maintain PaO2 > 50 mm Hg, or pulse oximeter saturation > 85%, and (c) characteristic chest radiographic findings within the first 24 h of life [27]. Regarding term neonates, various diagnostic criteria have been proposed [28,29,30,31]. We used the diagnostic criteria of Liu et al. [31]: (a) acute onset of the respiratory distress, (b) a perinatal insult, such as birth asphyxia, perinatal hypoxia/fetal distress [32], elective caesarean section etc., apart from early onset sepsis, (c) progressive respiratory distress occurring shortly after birth, including characteristic grunting respiration, retractions during inspiration, cyanosis, and reduced or absent breathing sounds, and (d) characteristic radiographic findings in chest x-ray.

In the study, vitamin K was administered intramuscularly to neonates immediately after birth. ROTEM was performed before surfactant administration. Neonates who were transfused with blood products, and neonates with suspected or confirmed sepsis were excluded.

As control group for the EXTEM (tissue factor-triggered extrinsic pathway) assay, we used 282 previously recruited healthy neonates [33]. As control group for the INTEM (ellagic acid activated intrinsic pathway), and FIBTEM (with platelet inhibitor cytochalasin D) assays, we recruited 102 healthy neonates. Healthy neonates were defined based on the EXTEM control group study [33].

### 2.3. Data Synthesis

Data on demographics, maternal and pregnancy history, maternal medication during pregnancy, prenatal administration of corticosteroid therapy, Apgar score in the first and fifth minute of life, cord pH, first hour pH and pH on admission, laboratory results on admission (white blood cell (WBC) count, neutrophils, hematocrit (Hct), nucleated red blood cells (NRBC), platelet (PLT) count, C-reactive protein (CRP)), chest radiographic findings, type and duration of ventilation and oxygen therapy, bleeding events, cerebral ultrasonographic findings, hospital stay, day of establishing full enteral feeding, and mortality were recorded. All neonates were followed up until hospital discharge, where brain and abdomen ultrasound scans were performed in all neonates weekly (as per our NICU protocol) and whenever there was clinical suspicion of bleeding or thrombosis.

Downes score and SNAP-PE SCORE (Score for Neonatal Acute Physiology-Perinatal Extension) were calculated for all recruited neonates on admission to the NICU [34,35,36,37].

ROTEM was performed for all recruited neonates using ROTEM Delta Analyzer (Tem Innovations GmbH, Munich, Germany). Arterial blood was drawn through a peripheral artery using a 23-gauge 0.6-mm needle and collected into a citrated tube (0.109 mol/L trisodium citrate) by a vacutainer system and stored at room temperature until testing. In <30 min, the citrated arterial blood was incubated for 2–5 min at 37 °C and was tested using the ROTEM analyzer. EXTEM, INTEM, and FIBTEM assays were performed. In the EXTEM assay, clot formation was induced by activation of the extrinsic coagulation pathway using 20 μL of 0.2 M calcium chloride solution (star-TEM reagent) and 20 μL of extrinsic activator—tissue thromboplastin (ex-TEM reagent, recombinant tissue factor and phospholipids). After the reagents were adequately mixed, 300 μL of citrated whole blood was added to the cup and the assay was running for at least 60 min after the completion of clot lysis at 30 min. Accordingly, in the INTEM assay, clot formation was induced by activation of the intrinsic coagulation pathway using 20 μL of 0.2 M calcium chloride solution (star-TEM reagent) and 20 μL of intrinsic activator [in-TEM reagent, partial thromboplastin phospholipid made of rabbit brain (chloroform extract), ellagic acid]. The FIBTEM assay consists of a modified EXTEM assay with the addition of a potent platelet inhibitor (cytochalasin D), which blocks platelet activation, shape change, and expression and activation of glycoprotein IIbIIIa (fibrinogen) receptors. In all assays, the following variables were measured: clotting time (CT, in seconds), exhibiting the time for the formation of a 2 mm clot in amplitude; clot formation time (CFT, in seconds), showing the time from CT until the achievement of a clot firmness of 20 mm; amplitude at 10 min (A10, in millimeters); maximum clot firmness (MCF, in millimeters), which is the final strength of the clot; and lysis index in 60′ (LI60, in percentage), which is the percentage of remaining clot stability in relation to MCF following the 60′ observation period after CT, which indicates the speed of fibrinolysis [24].

### 2.4. Statistical Analysis

Descriptive statistics were used to present the baseline characteristics and laboratory findings of the study and control groups. Absolute and proportional values for nominal variables and means with standard deviation (SD) or median with interquartile ranges (IQRs) were used according to the normality of the distribution for the numerical variables. The normality of distributions was checked with the Shapiro–Wilk test. Chi-square test was used for comparisons between categorical variables. The independent samples *t*-test and the non-parametric Wilcoxon–Mann–Whitney test for two group comparisons were used. Pearson’s r and Spearman’s rank correlation coefficients were used, according to the normality of distribution, to assess the existence of positive or negative correlation between Downes Score and SNAP-PE Score with ROTEM variables. Finally, to evaluate the independent impact of coagulation profile as reflected by ROTEM parameters on development of RDS, a multivariable logistic regression was performed with RDS as the dependent variable, and ROTEM parameters, prematurity, gender, BW, and fetal distress as independent variables. The regression model was run separately for each ROTEM parameter as a dependent parameter due to multicollinearity between the ROTEM parameters. SPSS 26 for Windows statistical package (SPSS Inc., Chicago, IL, USA) was used for analysis. For all tests, a *p*-value < 0.05 indicated statistical significance.

## 3. Results

Our sample consisted of 48 neonates (24 term and 24 preterm) (Median GA: 36 weeks (IQR: 34.25–38 weeks)) that developed RDS during the study period, and were compared with 282 healthy neonates (198 term and 84 preterm) previously recruited regarding the EXTEM assay [33], and 102 newly recruited healthy neonates (85 term and 17 preterm) regarding the INTEM and FIBTEM assays. General characteristics of the study and control groups are presented in Table 1, while clinical characteristics of the study group, as well hematological and biochemical parameters at baseline are shown in Table 2.

The Medians and IQRs for EXTEM, INTEM and FIBTEM parameters of the study group, as well as those of the control groups in term and preterm neonates are shown in Table 3. The comparison between groups exhibited significant prolonged EXTEM CT and CFT, decreased EXTEM A10, MCF, and LI60, prolonged INTEM CFT, and decreased INTEM A10 and MCF; prolonged FIBTEM CT, decreased FIBTEM A10 and MCF in both term and preterm neonates with RDS in comparison to healthy neonates were found (*p*-values < 0.001 except for FIBTEM CT in preterm neonates: *p*-value = 0.010).

Multivariable logistic regression analysis (Table 4) showed that RDS is significantly associated with longer EXTEM CT, lower EXTEM A10, MCF, and LI60, lower INTEM A10 and MCF, lower FIBTEM A10 and MCF when adjusted for gender, prematurity, BW, and fetal distress (*p*-values < 0.001).

No significant correlation was found between the Downes Score or SNAP-PE Score and ROTEM variables (Table 5).

The clinical outcomes of neonates with RDS are presented in Table 6. IVH observed in 18 of the neonates was grade I and no correlation was found between IVH and ROTEM parameters (Table 7). No severe bleeding events were recorded in the study population.

## 4. Discussion

In this study, we investigated the alterations of ROTEM variables in neonates with RDS, and examined the alterations of EXTEM, INTEM, and FIBTEM assays in neonates in comparison to healthy ones. In EXTEM, due to the extrinsic activation, initial thrombin generation and hence initial clotting, mainly depends on the activity of the coagulation factors VII, X, V, II, and fibrinogen. In INTEM, due to the intrinsic activation, such as the activated partial thromboplastin time, initial thrombin generation and clot formation depend on coagulation factors XII, XI, IX, VIII, X, V, II, and fibrinogen. Clot firmness in INTEM assay as well as in EXTEM assay reflects both fibrin and platelet contribution to the strength of the clot. In FIBTEM, clot formation and clot strength depend only on fibrin formation and fibrin polymerization, as the thrombocytes are blocked [38]. Our results showed a more hypocoagulable profile (prolongation of CT and CFT as well as decreased A10, meaning prolonged clot formation and smaller clot size at 10 min, respectively) with higher fibrinolytic potential (smaller LI60) in both term and preterm neonates with RDS compared to healthy neonates.

Neonates have a hemostatic deficit compared to older children and adults, but their hemostatic system is perfectly functionally balanced [39]. They have decreased levels of most coagulation factors, except for fibrinogen, factor V (FV), factor VIII (FVIII), and von Willebrand factor (vWF), compared to adults, which probably causes shorter coagulation time [40,41]. Moreover, neonates present impaired polymerization fibrin properties, which could cause diminished clot strength [41,42]. Finally, neonates have increased tissue-plasminogen activator (t-PA) levels and reduced levels of plasminogen activator inhibitor (PAI) and α2-antiplasmin, compared to adults, potentially leading to a more intense fibrinolytic activity [41,43].

Respiratory distress syndrome is characterized by diffuse atelectasis, high permeability due to epithelial injury, pulmonary edema, fibrin deposits forming hyaline membranes, and finally, right to left shunting of pulmonary blood flow [7,44,45,46]. The fourfold increase of epinephrine in neonates with RDS [47] can cause vasoconstriction and subsequently epithelial injury, which in turn leads to platelet agglutination and the formation of thrombi [48]. The damaged cells release in the extracellular space damage-associated molecular patterns (DAMPs), which are endogenous intracellular molecules, such as high mobility group box 1 (HMGB1), histones, purine metabolites, uric acid, and mitochondrial components [49,50]. DAMPs trigger inflammation through their detection by pattern recognition receptors (PRRs), such as Toll-like receptors, and also they trigger the formation of thrombi by inducing tissue factor expression on monocytes, elevating tissue factor procoagulant activity, and promoting platelet aggregation [50]. Apart from the increased platelet agglutination, the observed hypercoagulability may be the result of heparin co-factor deficiency, increased anti-heparin platelet activity, and an increased activator or a qualitative deficiency in neonatal fibrinogen [51,52,53]. The process of RDS leads to the consumption of coagulation factors. Firstly, FV and FVIII, as well as the platelets are depleted, followed by factors I and II. As the clotting continues, the fibrinolytic activity increases with the production of fibrin degradation products. The coagulation factors’ consumption and the antithrombin (AT) effect of the fibrin degradation products, finally, lead to hypocoagulability [54].

Brus et al. [9] have found increased TAT levels in preterm neonates with severe RDS compared to preterm neonates with mild/moderate RDS, while Yurdakok et al. [55] have found normal TAT levels in neonates with RDS in the first hours after birth. These findings suggest that in early stages of RDS increased clotting activity is not prominent. Moreover, Brus et al. [9] reported increased tissue-plasminogen activator (t-PA) levels in preterm infants with RDS in the first 6-12 h after birth, while Yurdakok et al. [55] found normal t-PA levels, but increased plasminogen activator inhibitor (PAI) levels in preterm neonates with RDS in their first 6 h of life. The variation in results might be due to the different stages of the RDS, pointing out the insufficiency that exists regarding the coagulation and fibrinolytic system in different stages of RDS. Moreover, both these studies [9,55] examined individual coagulation factors without being able to globally delineate the hemostatic profile of neonates with RDS.

Our study sample consisted mainly of moderate to late preterm and term neonates with mild/moderate RDS; the median hour of ROTEM examination was the fifth (IQR: 5–6.75) hour of life, and we found decreased clotting and increased fibrinolytic potential in these neonates. Our results are in accordance with the findings of Watkins et al. [56], and Go et al. [13]. Watkins et al. [56] compared the TEG parameters of 13 neonates with respiratory failure to 11 healthy preterm and term neonates. While R, RK, Angle, and MA were similar in both groups in the first hours of life, by 48 h there were significant differences. The reported prolongation of R and RK was related to plasma clotting factors and inhibitors, while the decrease of MA in neonates with respiratory failure was attributed to the loss of platelets. In the early stages of RDS, the epithelial injury stimulates the clotting system leading to hypercoagulability [57], but as Watkins et al. [56] showed, the consumption of the coagulation factors leads to hypocoagulability in the course of the disease. Our results probably depict this stage of coagulation factors’ consumption. Go et al. [13] showed that fibrinogen is affected in neonates with RDS (*p* < 0.001; β: −0.141). Fibrinogen depletion/dysfunction is also evident in neonates with RDS in our study as shown by the lower FIBTEM A10 and MCF which tend to be utilized as a surrogate of the plasma fibrinogen level [58]. Studies have shown that fibrinogen function is strongly correlated with FIBTEM MCF [58,59,60]. Tsantes et al. [59] showed that FIBTEM analysis can detect low fibrinogen availability/dysfunction associated with excessive bleeding in patients undergoing surgery even, when fibrinogen levels and conventional coagulation tests remain within reference range.

It is noteworthy that while 43.8% of the study population had fetal distress, after adjustment for fetal distress in the logistic regression analysis, RDS was found to be an independent factor for the same alterations in ROTEM variables as in fetal distress [24].

In our study, no correlation was found between the Downes and SNAP-PE Scores with the ROTEM variables’ alterations, probably because the study sample consisted mainly of neonates with mild/moderate RDS.

Finally, no correlation was found between IVH grade I and ROTEM variables’ alterations. Regarding the pathogenesis of IVH, coagulation disorders may amplify the hemorrhage [61], but IVH is mostly attributed to the combined immature vasculature of the germinal matrix, the fluctuations in cerebral blood flow (CBF), and the impaired cerebral autoregulation in critically ill neonates [62].

Our study has certain limitations. Our sample size was small, thus limiting the possibility to find significant results regarding several neonatal outcomes. Neonates <28 weeks GA were not included, which further impeded the investigation of outcomes, such as BPD. Another limitation is the fact that conventional coagulation tests were not performed at the same time to correlate with ROTEM parameters, due to the practice of minimal handling and the limited volume of residual blood. Finally, we did not have consecutive ROTEM measurements to investigate the alterations in ROTEM variables in the course of the disease.

## 5. Conclusions

Moderate/late preterm and term neonates with RDS present a hypocoagulable profile with hyperfibrinolytic potential compared to healthy neonates. ROTEM seems a potentially useful tool for identifying alterations regarding the hemostatic profile as well for detecting coagulation and fibrinolysis impairment in neonates with RDS. Further cohort studies are necessary to clarify the impact of RDS in the neonatal hemostasis as well as determine the role of ROTEM in diagnosis and management of coagulation disorders in neonates with RDS.

## Figures and Tables

**Table 1:** General characteristics of the neonates with RDS and the control groups.

	Neonates with RDS (n = 48)	Healthy Neonates—Group A (n = 282)	Healthy Neonates—Group B (n = 102)	Neonates with RDS vs. Group A *p*-Value	Neonates with RDS vs. Group B *p*-Value
Preterm neonates					
n (%)	24 (48.98)	84 (29.79)	17 (16)		
GA in weeks, Median (IQR)	35 (32.25–35.75)	34.5 (33–35)	35 (34–36)	0.636 *	0.443 *
BW in grams, Median (IQR)	2250 (1847.5–2867.5)	2.150 (1842.5–2.440)	2300 (2190–2475)	0.202 *	0.968 *
Delivery mode [CS, n (%)]	18 (75)	72 (85.7)	16 (94.1)	0.214 ^	0.207 ^
Gender [male, n (%)]	19 (79.2)	44 (52.4)	13 (76.5)	**0.019** ^	1.000 ^
**Term neonates**					
n (%)	24 (48.98)	198 (70.21%)	85 (80.19)		
GA in weeks, Median (IQR)	38 (38–39)	39 (37–39)	38 (38–39)	**0.039** *	0.053 *
BW in grams, Median (IQR)	3085 (2800–3415)	3.245 (2907.5–3492.5)	3250 (3000–3575)	0.245 *	0.077 *
Delivery mode [CS, n (%)]	19 (79.2)	78 (39.4)	34 (40)	**<0.001** ^	**0.001** ^
Gender [male, n (%)]	19 (79.2)	89 (44.9)	40 (47.1)	**0.002** ^	**0.006** ^

Abbreviations: BW, body weight; CS, caesarean section; GA, gestational age; IQR, interquartile range (difference between 75th and 25th percentiles); RDS, respiratory distress syndrome. * The non-parametric Mann–Whitney U test was used. ^ The chi–square test was used.

**Table 2:** Clinical characteristics of the neonates with RDS and laboratory findings at baseline (N = 48).

Clinical Characteristic	Values
Twin pregnancy, n (%)	3 (6.3)
Fetal distress, n (%)	21 (43.8)
Gestational diabetes, n (%)	4 (8.3)
Preeclampsia, n (%)	4 (8.3)
Placental abruption, n (%)	4 (8.3)
IUGR, n (%)	2 (4.2)
SGA, n (%)	2 (4.2)
ELSCS, n (% of all CS)	33 (89.19)
Prenatal steroids, n (%)	9 (18.4)
CHD, n (%)	3 (6.2)
Hour of life on admission, Median (IQR)	5 (5–6.75)
Ph on admission, Mean (±SD)	7.32 (0.07)
Air leak syndrome, n (%)	12 (24.6)
PO_2_/FiO_2_ (mmHg), Median (IQR)	230.4 (155.67–315)
Downes score, Mean (±SD)	2.35 (1.72)
SNAP-PE score, Mean (±SD)	5.71 (5.54)
WBC (K/μL), Mean (±SD)	17.06 (6.09)
Neu (K/μL), Mean (±SD)	11 (4.82)
NRBC (M/μL), Mean (±SD)	4.48 (0.64)
Hct (%), Mean (±SD)	45.8 (6.34)
PLT (K/μL), Mean (±SD)	260.57 (70.94)
CRP (mg/L), Median (IQR)	1.5 (0.7–2.2)

Abbreviations: CHD, congenital heart disease; CRP, c reactive protein; CS, caesarean section; ELSCS, elective lower segment caesarean section; FiO_2_, fraction of inspired oxygen; Hct, Hematocrit; IQR, interquartile range (difference between 75th and 25th percentiles); IUGR, intrauterine growth restriction; Neu, neutrophils; NRBC, nucleated red blood cells; PLT, platelet count; PO_2_, partial pressure of oxygen; RDS, respiratory distress syndrome; SD, standard deviation; SGA, small for gestational age; SNAP-PE, Score for Neonatal Acute Physiology-Perinatal Extension; WBC, white blood cell count.

**Table 3:** Comparison of ROTEM parameters between healthy neonates and neonates with RDS.

ROTEM Variable	EXTEM			INTEM			FIBTEM		
	Healthy Term Neonates (N = 198)	Term Neonates with RDS (N = 24)	*p*-Value *	Healthy Term Neonates (N = 85)	Term Neonates with RDS (N = 24)	*p*-Value *	Healthy Term Neonates (N = 85)	Term Neonates with RDS (N = 24)	*p*-Value *
CT (sec)	41 (36–51)	63 (55–74)	**<0.001**	202 (184–223.5)	215 (182–243)	0.268	48 (41.5–56.5)	59.5 (50.25–70.75)	**<0.001**
CFT (sec)	58 (53–63)	122 (97–138)	**<0.001**	75 (63.5–90.5)	89 (81–108)	**<0.001**	263 (3.77–679)		
A10 (mm)	65 (59.75–69)	44 (42–47)	**<0.001**	54 (50–57)	48 (46–50)	**<0.001**	14 (12–17)	9 (8–11)	**<0.001**
MCF (sec)	66 (60–71)	53 (50–55)	**<0.001**	59 (55–62)	53 (50–55)	**<0.001**	16 (13–19)	10.5 (9–13)	**<0.001**
LI60 (%)	97 (95–99)	94 (92–95)	**<0.001**	93 (90–95)	93 (92–94)	0.963	100	100 (97–100)	0.189
	**Healthy preterm neonates (N = 84)**	**Preterm neonates with RDS (N = 24)**		**Healthy preterm neonates (N = 17)**	**Preterm neonates with RDS (N = 24)**		**Healthy preterm neonates (N = 17)**	**Preterm neonates with RDS (N = 24)**	
CT (sec)	44 (37–51)	58 (49–64)	**<0.001**	202 (189–218)	198 (175–237)	0.980	48 (44–53)	57 (46–84.25)	0.010
CFT (sec)	57.5 (52–64.75)	101 (92–130)	**<0.001**	58 (51–76)	82 (65–103)	**<0.001**	118 (1.86–242)		
A10 (mm)	62 (57.25–68)	45 (42–52)	**<0.001**	58 (55–63)	47 (45–52)	**<0.001**	17 (14–20)	10 (7–13)	**<0.001**
MCF (sec)	64 (57.25–70.75)	51 (49–57)	**<0.001**	62 (59–67)	53 (48–57)	**<0.001**	19 (15–23)	11 (7.75–14)	**<0.001**
LI60 (%)	96 (93–100)	91 (91–94)	**<0.001**	92.5 (90.75–94.25)	92 (90–94)	0.382	100 (99–100)	100 (97.5–100)	0.948

Abbreviations: A10, clot strength at 10 min (mm); CFT, clot formation time (seconds); CT, clotting time (seconds); EXTEM, extrinsically activated TEM; FIBTEM, fibrin-based extrinsically activated TEM; INTEM, intrinsically activated TEM; LI60, lysis index at 60 min (%); MCF, maximal clot firmness (mm); ROTEM, rotational thromboelastometry. All presented data are medians (25th–75th percentile). * The non-parametric Mann Whitney U test was used.

**Table 4:** Results of the multivariable logistic regression analysis for RDS as the dependent variable, with ROTEM parameters, age (preterm vs. postterm), sex, BW, and perinatal hypoxia/fetal distress as independent variables.

ROTEM Parameters	OR	95% CI	*p*-Value
EXTEM CT (sec)	1.087	1.050–1.126	**<0.001**
EXTEM CFT (sec)	990.825	0.000–2.603 × 10^124^	0.961
EXTEM A10 (mm)	0.778	0.716–0.845	**<0.001**
EXTEM MCF (mm)	0.866	0.818–0.916	**<0.001**
EXTEM LI60 (%)	0.846	0.774–0.925	**<0.001**
INTEM CT (sec)	0.996	0.979–1.013	0.649
INTEM CFT (sec)	1.012	0.994–1.031	0.192
INTEM A10 (mm)	0.840	0.752–0.939	**0.002**
INTEM MCF (mm)	0.786	0.685–0.904	**<0.001**
INTEM LI60 (%)	0.869	0.764–1.050	0.174
FIBTEM CT (sec)	1.002	0.995–1.009	0.550
FIBTEM CFT (sec)	0.827	0.000–1.7396 × 10^34^	0.996
FIBTEM A10 (mm)	0.511	0.354–0.739	**<0.001**
FIBTEM MCF (mm)	0.594	0.448–0.787	**<0.001**
FIBTEM LI60 (%)	1.067	0.912–1.248	0.420

Abbreviations: CI, confidence interval; CT, clotting time (seconds); CFT, clot formation time (seconds); A10, clot strength at 10 min (mm); EXTEM, extrinsically activated TEM; FIBTEM, fibrin based activated TEM; INTEM, intrinsically activated TEM; IVH, intraventricular hemorrhage; LI60, lysis index at 60 min (%); MCF, maximal clot firmness (mm); OR, Odds ratio; RDS, respiratory distress syndrome; ROTEM, rotational thromboelastometry.

**Table 5:** Downes Score and SNAP-PE Score in correlation with alterations in ROTEM variables.

	CT (sec)	CFT (sec)	A10 (mm)	MCF (mm)	LI60 (%)
EXTEM					
**Downes Score**					
Correlation coefficient	−0.062 *	−0.163 *	0.186 *	0.262 *	0.226 *
*p*-value	0.688	0.29	0.227	0.086	0.14
**SNAP-PE Score**					
Correlation coefficient	−0.055 *	−0.261 *	0.067 *	0.036 *	0.052 *
*p*-value	0.714	0.077	0.654	0.811	0.727
**INTEM**					
**Downes Score**					
Correlation coefficient	−0.024 *	−0.192 *	0.155 *	0.163 *	0.053 **
*p*-value	0.877	0.212	0.316	0.29	0.732
**SNAP-PE Score**					
Correlation coefficient	−0.103 *	−0.245 *	0.122 *	0.094 *	−0.153 **
*p*-value	0.489	0.096	0.414	0.531	0.304
**FIBTEM**					
**Downes Score**					
Correlation coefficient	−0.113 *	0.483 *	0.022 *	0.172 **	−0.128 **
*p*-value	0.466	0.517	0.888	0.264	0.409
**SNAP-PE Score**					
Correlation coefficient	0.074 *	−0.392 *	−0.048 *	−0.134	0.084 **
*p*-value	0.623	0.442	0.749	0.376	0.575

Abbreviations: CT, clotting time (seconds); CFT, clot formation time (seconds); A10, clot strength at 10 min (mm); EXTEM, extrinsically activated TEM; FIBTEM, fibrin based activated TEM; INTEM, intrinsically activated TEM; LI60, lysis index at 60 min (%); MCF, maximal clot firmness (mm); ROTEM, rotational thromboelastometry; SNAP-PE, Score for Neonatal Acute Physiology-Perinatal Extension. * The Pearson’s r correlation coefficient was used. ** The Spearman’s rho correlation coefficient was used.

**Table 6:** Clinical outcomes of the neonates with RDS.

Clinical Outcome	Values
IVH grade I, n (%)	18 (37.5)
Oxygenation therapy, days, Median (IQR)	4 (2–6.62)
Full enteral nutrition, days, Median (IQR)	8 (5.75–9.25)
Hospitalization days, Median (IQR)	12.5 (8.25–21)
BPD, n (%)	2 (4.2)
Death, n (%)	1 (2)

Abbreviations: BPD, bronchopulmonary dysplasia; IQR, interquartile range (difference between 75th and 25th percentiles); IVH, intraventricular hemorrhage; RDS, respiratory distress syndrome.

**Table 7:** ROTEM variables in neonates with RDS in correlation to IVH.

ROTEM Variables	IVH ^			
		Neonates with IVH (n = 18)	Neonates without IVH (n = 30)	*p*-Value
EXTEM CT (sec)	Median (IQR)	58 (49–64.5)	59.5 (52.75–67)	0.427 **
EXTEM CFT (sec)	Median (IQR)	107 (95.5–129)	110.5 (92.5–137)	1.000 **
EXTEM A10 (mm)	Mean (±SD)	45 (5.969)	46.04 (7.721)	0.233 *
EXTEM MCF (sec)	Median (IQR)	50 (49–54.5)	52.5 (49.25–58.75)	0.388 **
EXTEM LI60 (%)	Median (IQR)	93 (91–95)	93 (91–95)	0.894 **
INTEM CT (sec)	Mean (±SD)	211.44 (34.398)	211.84 (43.066)	0.403 *
INTEM CFT (sec)	Median (IQR)	84 (79.5–98.75)	96 (67–114.5)	0.454 **
INTEM A10 (mm)	Mean (±SD)	47.88 (5.807)	47.88 (7.628)	0.239 *
INTEM MCF (sec)	Mean (±SD)	52.81 (5.063)	53.12 (6.133)	0.514 *
INTEM LI60 (%)	Median (IQR)	92.5 (90.25–94)	93 (91–94)	0.936 **
FIBTEM CT (sec)	Median (IQR)	63 (51.25–82.75)	56 47.5–80.5)	0.356 **
FIBTEM CFT (sec)	Median (IQR)	1.45 (1.68–490.22)	286.34 (1.68–571)	0.800 **
FIBTEM A10 (mm)	Mean (±SD)	10.06 (4.389)	10.56 (3.675)	0.314 *
FIBTEM MCF (sec)	Median (IQR)	9.5 (7–16.25)	11 (8–14)	0.376 **
FIBTEM LI60 (%)	Median (IQR)	100 (97.25–100)	100 (98–100)	0.987 **

Abbreviations: CT, clotting time (seconds); CFT, clot formation time (seconds); A10, clot strength at 10 min (mm); EXTEM, extrinsically activated TEM; FIBTEM, fibrin based activated TEM; INTEM, intrinsically activated TEM; IVH, intraventricular hemorrhage; LI60, lysis index at 60 min (%); MCF, maximal clot firmness (mm); RDS, respiratory distress syndrome; ROTEM, rotational thromboelastometry. ^ All neonates with IVH had grade I IVH. * The independent sample *t*-test was used. ** The non-parametric Mann–Whitney U test was used.

## Data Availability

Available upon request.
